# Roles of Secretory Immunoglobulin A in Host-Microbiota Interactions in the Gut Ecosystem

**DOI:** 10.3389/fmicb.2022.880484

**Published:** 2022-06-02

**Authors:** E. Daniel León, M. Pilar Francino

**Affiliations:** ^1^Department of Genomics and Health, Fundación Para el Fomento de la Investigación Sanitaria y Biomédica de la Comunitat Valenciana (FISABIO), Valencia, Spain; ^2^CIBER en Epidemiología y Salud Pública, Madrid, Spain

**Keywords:** gut microbiota, microbiome, secretory IgA, immune system, cross-species reactivity

## Abstract

In the gastrointestinal tract (GIT), the immune system interacts with a variety of microorganisms, including pathogens as well as beneficial symbionts that perform important physiological functions for the host and are crucial to sustain intestinal homeostasis. In normal conditions, secretory immunoglobulin A (SIgA) is the principal antibody produced by B cells in the GIT mucosa. Polyreactivity provides certain SIgA molecules with the ability of binding different antigens in the bacterial surface, such as O-antigens and teichoic acids, while cross-species reactivity allows them to recognize and interact with different types of bacteria. These functions may be crucial in allowing SIgA to modulate the complex gut microbiota in an efficient manner. Several studies suggest that SIgA can help with the retention and proliferation of helpful members of the gut microbiota. Gut microbiota alterations in people with IgA deficiency include the lack of some species that are known to be normally coated by SIgA. Here, we discuss the different ways in which SIgA behaves in relation to pathogens and beneficial bacteria of the gut microbiota and how the immune system might protect and facilitate the establishment and maintenance of certain gut symbionts.

## Introduction

The GIT of mammals is inhabited by large numbers of microorganisms, mainly bacteria belonging to hundreds of different species. In humans and mice, the majority of the bacterial species in the GIT microbiota belong to two main phyla: Firmicutes and Bacteroidetes (Krych et al., 2013). The GIT microbiota performs numerous beneficial functions for the host, related to nutrient production, lipid and carbohydrate homeostasis, synthesis of hormones and neurotransmitters, and modulation of the immune system, among others. Despite these important functions, the specific composition of the GIT microbiota varies greatly among healthy individuals, indicating a degree of functional redundancy among different GIT bacteria (Turnbaugh et al., 2009; Vaishampayan et al., 2010). However, substantial alterations of GIT microbiota composition can result in a variety of diseases, including metabolic and immune disorders (Francino, 2017; Thursby and Juge, 2017; Moran et al., 2019).

Given the high density of microbial colonization, the GIT is an important site where the host immune system and microorganisms interact. The GIT bacteria reside mainly in the intestinal cavity, or lumen, and have only limited contact with the surrounding mucosal epithelium, from which they are separated by mucus (Johansson et al., 2015). The GIT mucosa epithelium and the underlying layer of loose connective tissue, the lamina propria, contain a sophisticated and specialized mucosal immune system. This system involves inductive sites, at which antigens sampled from the mucosal surface stimulate naïve T and B cells, as well as effector sites, where various effector cells perform their actions, such as antibody production and secretion. Inductive sites involve mainly the gut-associated lymphoid tissue and the mesenteric lymph nodes that drain the mucosa, both of which contain lymphocyte aggregates known as lymphoid follicles; in the gut-associated lymphoid tissue, lymphoid follicles agglomerate in the Peyer’s patches (PPs) of the small intestine or occur in isolation, with the density of isolated follicles increasing distally. Effector sites involve the mucosal epithelium and the lamina propia (Brandtzaeg et al., 2008; Kobayashi et al., 2019; Sun et al., 2020).

Both innate and adaptive responses are involved in the interaction between microorganisms and the host immune system and therefore in shaping the GIT microbiota (Cerutti and Rescigno, 2008; Bonilla and Oettgen, 2010). The innate immune system is the first barrier vs. all exogenous molecules that enter the body, but its response is poorly specific (Hillion et al., 2020). In contrast, the adaptive immune system recognizes antigens through specific surface receptors expressed on T and B cells. The cells of the innate immune system sense microorganisms or their metabolic products and elicit several responses (Round and Mazmanian, 2009; Thaiss et al., 2016). Intestinal epithelial cells also encode a variety of receptors for ligands of microbial origin. Engagement of receptors in innate immune and epithelial cells results in the production of cytokines, which influence the differentiation of the naïve T cells of the adaptive immune system. These cells can differentiate into regulatory cells (Tregs) or into helper cells, including Th1, Th2, and Th17 (Romagnani, 2006; Platt and Mowat, 2008). Tregs possess a plethora of anti-inflammatory roles and can downregulate the activation and development of the different Th types (Groux et al., 1997; Romagnani, 2004), which in turn play a variety of specific roles in shaping the immune response (Mosmann et al., 1986; von der Weid et al., 2001). Therefore, an aberrant microbial colonization of the GIT can produce an imbalance among the different types of T cells, and the consequent immune deregulation can generate a variety of pathological outcomes, ranging from atopy to autoimmune disease (Wills-Karp et al., 2001; Noverr and Huffnagle, 2005; Rook and Brunet, 2005, 2016; Francino, 2014; Shu et al., 2019; Tirone et al., 2019).

Among other things, activated Th cells induce B cells to produce antibodies, also called immunoglobulins (Igs). The highest levels of antibodies are generated and secreted at mucosal surfaces of the gastrointestinal, urogenital, and respiratory tracts, whereas systemic antibodies in the bloodstream are found in lower concentrations (Quan et al., 2001; Li et al., 2019). Importantly, the gut contains the largest populations of plasma cells (PCs), the activated B cells that produce antibodies, and the antibodies they produce are transported into the gut lumen. Immunoglobulin A (IgA), IgM, and IgG are antibodies present in the intestine with the capacity to coat different bacteria (Bunker and Bendelac, 2018; Janzon et al., 2019). IgG coats a reduced proportion of the gut microbiota. It has been shown that IgG is not produced continuously in the gut, since it is mainly activated by antigens present in pathogens such as *Haemophilus* that can cross the intestinal barrier. On the other hand, IgA and IgM coat similar members of the microbiota present in the lumen. However, IgM occurs at a concentration nearly 100 times lower than that of IgA (Haneberg and Aarskog, 1975; Janzon et al., 2019). To date, only a few studies have focused on the role of IgG and IgM antibodies in host-microbiota symbiosis. For these reasons, we will concentrate here in the diverse interactions between IgA and the gut microbiota and will evaluate their potential contribution to the generation of a symbiotic environment in the gut.

## Basic IgA Biology

Immunoglobulin A is present in monomeric form in the blood, but only dimeric and other minor polymeric forms are found in mucosal secretions such as colostrum and barrier surfaces such as the intestinal mucosa (Almogren et al., 2007; Yel, 2010). Only polymeric IgA forms can be actively transported across mucosal surfaces for secretion. When polymeric IgA produced by PCs binds the polymeric Ig receptor (pIgR) expressed by epithelial cells, secretory IgA (SIgA) is formed (Pabst and Slack, 2020). The IgA binds to pIgR in the basolateral surface of epithelial cells and is internalized into endosomes. Next, it is transported in vesicles to the apical surface. Subsequently, it is proteolytically cleaved and the extracellular fragment called Secretory Component (SC) is liberated with the IgA ligand. The SC is covalently bound to the antibody portion and constitutes an integral part of the SIgA complex.

Approximately 3 g per day of SIgA are produced in adult humans, which is more than the daily production of all other Ig isotypes combined (Mostov, 1994; Kaetzel et al., 2017). Remarkably, the secretion of SIgA takes place only at very low levels in the intestinal lumen of germ-free mice, whereas colonization of their GIT is rapidly followed by the detection of normal values of SIgA. Thus, mucosal secretion of SIgA is partially controlled by the microbiota (Hapfelmeier et al., 2010; Kaetzel, 2014). In epithelial cells, extracellular receptors such as TLR2 and intracellular receptors such as the nucleotide-binding oligomerization domain-containing protein 2 (NOD2) have the capacity to recognize components of the bacterial cell wall or metabolites released by the gut microbiota and trigger the NFκB pathway. This pathway induces an increase of the phosphorylation of tight junction proteins facilitating the expression of pIgR (Mathias and Corthésy, 2011; Kaetzel, 2014), and therefore determining the rate of SIgA production and secretion.

### T-Cell-Dependent and T-Cell-Independent SIgA Responses

Like all antibodies, IgA is produced by plasma B cells, which collectively are capable of expressing a huge repertoire of distinct IgA molecules due to various mechanisms of sequence diversification. IgA and other antibodies are the unattached form of the antigen-binding B-cell receptor (BCR) that is released from B cells. Each primary B cell expresses a BCR involving an immunoglobulin molecule formed by two heavy (IgH) and two light (IgL) chains, with antigen-binding variable (V) regions located at their N-terminal ends. V regions are encoded by a combination of variable (V), diversity (D), and joining (J) gene segments assembled through V(D)J recombination during B cell development in the bone marrow, from a large number of different V, D, and J segments present in the germline. In addition, junctions between V, D, and J regions are diversified through deletions or additions of non-templated nucleotides during this recombination process (Alt et al., 2013). BCRs can further diversify through somatic hypermutations (SHM) during the process of affinity maturation toward a specific antigen. During affinity maturation, B cells that have undergone mutations providing increased affinity of the BCR for the antigen are positively selected (Di Noia and Neuberger, 2007; Victora and Nussenzweig, 2012).

In response to infection or immunization, antigen-activated B cells undergo antigen-driven BCR affinity maturation in a T-cell dependent process. This process occurs in germinal centers, specialized microstructures that form transiently in lymphoid organs and where long-lived antibody-secreting PCs and memory B cells are produced (Victora et al., 2010). Recent work has demonstrated that IgAs that recognize GIT microbiota members can also undergo such T-cell-dependent, antigen-driven affinity maturation. In this case, affinity-maturation takes place within permanent germinal centers located in gut-associated lymphoid structures such as PPs (Chen et al., 2020; Nowosad et al., 2020). Chen et al. (2020) were able to elucidate the repertoire of BCR sequences present in the germinal centers of PPs in mice colonized by an SPF (specific pathogen-free) microbiota. They showed that this repertoire was enriched across individual mice in particular types of heavy- and light-chain V segments containing specific SHMs. Although some of these BCR sequences were also enriched in germ-free mice, others were dependent on the presence of the SPF microbiota. Microbiota-dependent BCR types were shown to react against microbial glycans and to lose affinity towards these upon SHM reversion. Furthermore, microbiota-dependent BCR types became enriched in germ-free mice upon transfer of SPF mice feces. These results demonstrate that microbiota antigens can drive affinity maturation of BCRs in the PP germinal centers of the mouse gut. However, germ-free mice PP germinal centers were also enriched in some of the most frequent BCR sequence types present in SPF mice. Moreover, one of the BCR types present in both SPF and germ-free mice was shown not to react against an array of bacterial glycans, suggesting that it was directed toward a different type of antigen, presumably of non-bacterial origin.

Further, Nowosad et al. (2020) showed that B cell clonal selection and antigen-driven maturation in germinal centers occur at rates that depend on the type of microbiota present in the GIT. They analyzed B cell clones in germ-free mice and in mice colonized by an SPF microbiota or by a low diversity microbial consortium, i.e., a consortium of low species richness containing only 12 mouse gut bacterial strains. In accordance with the results of Chen et al. (2020), they identified B cell selection resulting in recurrent BCR types across animals in germ-free as well as microbiota-colonized mice. Remarkably, selection occurred at a much faster rate in germ-free mice than in SPF mice, and at an intermediate rate in mice colonized by the low diversity consortium. Also in accordance with Chen et al. (2020), antibodies encoded by B cells selected in the presence of GIT microbiota displayed a higher affinity to bacterial antigens than their unmutated precursors. In contrast, selection in germinal centers in germ-free conditions yielded antibodies whose specificity could not be determined, as they failed to react toward a series of common self, bacterial, and dietary antigens.

On the other hand, observations in experimental animal models demonstrate the presence of intestinal mucosal IgA in the absence of T cells, as was first shown in T-cell-deficient mice (Macpherson et al., 2000). Rather than occurring in germinal centers, T-cell-independent class switching to IgA seems to occur mainly within the gut lamina propria, where it may be promoted by dendritic cells or innate lymphoid cells (Cerutti, 2008; Cerutti and Rescigno, 2008; Lycke and Bemark, 2017; Pabst and Slack, 2020). Nevertheless, T cell help has been shown to be the major limiting factor for production of affinity-matured IgAs (Biram et al., 2019), so that a lack of SHMs can be considered indicative of a T-cell-independent response (Pabst and Slack, 2020).

Although it is clear that both T-cell-dependent and independent IgA responses can be generated, the contribution of each mechanism to IgA production and function in the GIT of mice and humans is subject to controversy, as their relative roles in both infection control and microbiota homeostasis remain undefined. On one hand, the majority of IgA-secreting PCs in the intestine of humans and aged mice are highly mutated and therefore likely to have been produced in a T-cell dependent manner (Barone et al., 2011; Gibbons and Spencer, 2011; Lindner et al., 2012). On the other, experiments in T-cell deficient mice showed that IgA coating of GIT microbiota species having inflammatory and invasive potential was significantly reduced, while that of other taxa, such as *Lactobacillus*, was increased (Palm et al., 2014). Indeed, several lines of evidence suggest that the high levels of affinity that characterize T-cell-dependent, affinity-matured SIgAs are essential for the efficient cross-linking and coating of potential pathogens, such as *Helicobacter*, *Salmonella*, and *Escherichia coli* (Kawamoto et al., 2012; Palm et al., 2014; Moor et al., 2017). Furthermore, in neonatal mice, both T-cell-dependent and independent IgA processes have been shown to participate in the production of IgAs (Mu et al., 2021). To investigate IgA induction in neonatal mice, Mu et al. (2021) analyzed mouse pups breastfed by immunodeficient mothers, showing that IgAs were induced by bacteria present in breastmilk, such as *Lactobacillus reuteri*. These IgAs stimulated by the maternal microbiota did not have the ability to cross-react vs. common enteric pathogens. IgA production was dependent on neonatal T cells, but was enhanced by a T cell–independent component involving type 3 innate lymphoid cells in the lamina propria of the small intestine (Mu et al., 2021).

### Canonical and Noncanonical SIgA-Bacteria Binding

Secretory immunoglobulin A-microbiota interactions can be carried out in two different ways, through canonical and noncanonical binding. The first involves Fab-region-dependent binding through complementarity determining regions (CDRs), while the second excludes CDR interactions and occurs through glycans associated to SIgA amino acids (Mathias and Corthésy, 2011;van de Bovenkamp et al., 2018; Pabst and Slack, 2020). Antibodies are highly glycosylated with different types of glycan residues attached in different regions of the molecule (van de Bovenkamp et al., 2018; Pabst and Slack, 2020). Moreover, the types of glycans present appear to vary among and within individuals depending on factors such as inflammatory status and rate of SIgA production (Pabst and Slack, 2020). Interestingly, van de Bovenkamp et al. (2018) showed that SHM of the BCR locus can affect glycosylation patterns. When SHM occurs, variable domains of the antibody are susceptible to acquire consensus amino acid motifs for N-glycosylation. van de Bovenkamp et al. (2018) reported that N-glycosylation sites in the Fab arms were mainly introduced at positions, which can influence antigen binding and affinity, near the CDRs and at the loop between the second and third CDR.

It is possible that cooperation between canonical and non-canonical binding contributes to stabilizing bacterial–SIgA interactions. Noncanonical binding alone may also be sufficient to ensure cross-linking between two bacteria. This has been shown in the case of interactions between SIgA glycans and bacterial adhesins (Sauer et al., 2016).

## SIgA-Microbiota Interactions

The GIT microbiota is a very complex assemblage and different members are likely to elicit IgA responses of variable type and intensity. Early studies (Crabbé et al., 1968; Moreau et al., 1978) showed that colonization of axenic mice by specific strains from the microbiota of conventionally raised mice resulted in different responses. Strains belonging to the Gram-positive genera *Lactobacillus*, *Streptococcus*, *Eubacterium*, *Actinobacillus*, *Micrococcus*, *Corynebacterium*, and *Clostridium* elicited a poor IgA response, whereas the Gram-negative *Bacteroides* and *Escherichia* induced a strong IgA response, no matter if they were alive or dead. More recently, Yang et al. (2020) monocolonized germ-free mice with dominant bacteria of the principal phyla of the human gut, i.e., Firmicutes, Bacteroidetes, Actinobacteria, and Proteobacteria. *Bacteroides ovatus* was the principal inducer of IgA production in the large intestine by the T-cell-dependent B-cell-activation pathway. Bacteria such as *Clostridium bolteae*, *Collinsella aerofaciens*, *Ruminococcus gnavus*, and *E. coli*, among others, triggered a poor IgA production. They also observed that heat-killed *B. ovatus* elicited a poor IgA response in *E. coli* pre-colonized mice, while viable *B. ovatus* carried a stronger response, contradicting earlier studies. Also, as mentioned above, Mu et al. (2021) showed that IgAs were induced in neonatal mice by a *Lactobacillus reuteri* present in the mothers’ breastmilk.

Numerous other studies have investigated the targets of SIgA by cell sorting of SIgA-coated bacteria in feces followed by 16S ribosomal RNA (rRNA) gene amplicon sequencing (IgA-Seq). In this approach, fecal bacteria are labeled with fluorescent anti-IgA antibodies and Fluorescence-Activated Cell Sorting (FACS) is used to separate those bacterial cells that are coated with IgA from those that are not. Then, 16S rRNA amplicon sequencing can be performed separately on IgA^+^ and IgA^−^ fractions. These studies have revealed that SIgA binds a highly diverse fraction of the human GIT microbiota, including members of its major constituent phyla, although specific bacterial taxa are found to be enriched in the SIgA-coated fraction (D'Auria et al., 2013; Palm et al., 2014; Bunker et al., 2015; Kau et al., 2015; Planer et al., 2016; Magri et al., 2017). There has been no overall consistency across studies regarding which specific bacteria were enriched, but enrichment of some taxa, such as those belonging to the prevalent Firmicutes family Lachnospiraceae, has been commonly detected (Sterlin et al., 2020). Lack of consistency may be related to differences in the IgA-Seq protocols implemented across laboratories and the indices employed to analyze the resulting data. A recent work has proposed a benchmarked IgA-Seq protocol and novel approaches to infer the IgA-binding probabilities of different taxa that will likely facilitate the comparison of results across laboratories (Jackson et al., 2021). Other limitations of this approach include the fact that IgA-Seq does not quantify the affinity of IgA antibodies directly, but rather provides information on the relative IgA-binding of different taxa. Other techniques, such as fluorescence *in situ* hybridization or enzyme-linked immunosorbent assays, are required to elucidate the actual affinities of host IgA for specific taxa (Jackson et al., 2021). Beyond IgA-Seq, the isolation by FACS of IgA-coated bacteria has allowed further insights into the interactions between IgA and the GIT microbiota. Importantly, mice colonized with SIgA-coated gut bacteria showed increased susceptibility to colitis (Palm et al., 2014) and enhanced diet-dependent enteropathy (Kau et al., 2015) compared with animals colonized with noncoated bacteria. These observations show that SIgA-coating is linked to distinct functional properties of gut bacteria. Differences in SIgA-microbiota binding have also been shown to relate to intestinal location, with SIgA-bound bacteria being more frequent in the small intestine (40%–80%) in comparison to the colon (10%–30%), and in mucus in comparison to the gut lumen (Palm et al., 2014; Bunker et al., 2015; Kau et al., 2015; Fadlallah et al., 2018).

Further, experiments in which microbiota members were exposed to different types of IgAs isolated from the GIT of adult humans revealed that some species were not bound by any of the antibodies, whereas others were targeted by one or several (Kabbert et al., 2020). The specific antigens bound by SIgA in the diversity of GIT bacteria and the underlying mechanisms of bacterial recognition and binding are still poorly understood. It is known that SIgA targets some important surface-exposed antigens of GIT bacteria, including glycans such as O-antigens, polysaccharide capsules, and teichoic acids. But, currently, no data are available regarding the affinity with which intestinal SIgA binds these molecules *in vivo*. Recently, Rollenske et al. (2021) mapped the collection of SIgAs produced in mice in response to colonization by a single *Escherichia coli* strain. They identified IgAs targeting a variety of bacterial antigens, including cell wall lipopolysaccharides (LPS), type I fimbriae, and outer membrane proteins (OmpW and OmpC). Some of these IgAs were polyreactive whereas most were antigen-specific. Expression of the different IgAs *in vivo* often resulted in lowered expression of the recognized antigen, as well as up- or downregulation of a variety of other genes. Depending on the antigen targeted, and the effects on gene transcription, different IgAs had distinct effects on the function and metabolism of the *E. coli* strain, relating to carbon-source uptake, bacteriophage susceptibility, or membrane integrity. Furthermore, IgAs targeting the same antigen could result in different effects depending on their particular epitope specificity. However, the downregulation of bacterial motility was an outcome common to all IgAs capable of bacterial surface coating.

On the other hand, the relevance of the less specific Fab-independent binding of SIgA to members of the microbiota is still under discussion. It has been proposed that the interaction between SIgA N-glycans and the peptidoglycan cell wall envelope of the Gram-positive symbionts that sustain intestinal homeostasis is mainly responsible for the recognition of these bacteria. Mathias and Corthésy (2011) showed discrepancies in the patterns of interaction through Fab-independent binding between Gram-positive and Gram-negative bacteria. They used a mouse hybridoma-derived reconstituted SIgA with a known specificity for *Shigella flexneri*, a Gram-negative pathogen, to prove the role of glycan residues in the interaction with the microbiota. Fluorescently labeled SIgA, free SC and their deglycosylated counterparts were combined with *Lactobacillus*, *Bifidobacterium*, *E. coli*, and *Bacteroides* strains. While both Gram-positive and Gram-negative bacteria were almost fully covered by glycosylated SIgA or free SC, the deglycosylated versions were less successful in covering Gram-positive bacteria. On the other hand, in Gram-negative bacteria, SIgA, free SC or their deglycosylated counterparts showed similar patterns of bacterial coating, suggesting the existence of other molecular mechanisms related with SIgA-Gram-negative bacteria interactions, likely involving LPS.

### Cross-Species Reactivity

Given the large number of different species present in the GIT microbiota, generating IgAs specific to single bacteria might be extremely costly for the host immune system. However, recent work has uncovered that a single monoclonal IgA can react with a defined variety of GIT microbiota species (Palm et al., 2014; Bunker et al., 2017; Kabbert et al., 2020). Various terms have been coined to describe the fact that one monoclonal Ig can bind several different members of the GIT microbiota, including “cross-specificity,” “polyspecificity,” and “cross-species reactivity.”

The mechanistic explanation of cross-species reactivity is unclear. It may be related to classical polyreactivity, the ability of an antibody to bind structurally unrelated antigens. Polyreactivity is typically associated with non-affinity-matured antibodies produced in germline configuration. It has indeed been shown that some monoclonal IgAs that show cross-species reactivity are also typically polyreactive, as they can bind a variety of antigens, such as LPS, CpG, and others. However, cross-species reactivity could also be due to IgAs targeting structures conserved across diverse species, such as common glycan or peptide motifs (Rollenske et al., 2018; Bunker et al., 2019; Kabbert et al., 2020; Sterlin et al., 2020).

In this respect, Bunker et al. (2017) investigated the specificity of IgAs produced by individual PCs from young mice toward the GIT microbiota. Most of these IgAs were capable of binding microbiota species with low to moderate affinity and showed cross-species reactivity, targeting many members of the Proteobacteria but largely excluding those of Bacteroidetes and Firmicutes, the predominant phyla in the colon. When tested against a battery of antigens, most of the IgAs were also found to be polyreactive and to be able to bind several antigens of bacterial origin, such as flagellin, LPS, and various glycans. This suggested that the capacity to recognize various antigens contributed to the observed cross-species reactivity. In addition, Bunker et al. (2017) analyzed the effect of somatic mutations on cross-species reactivity. To this aim, they reverted to germline configuration 21 highly mutated IgAs from the small intestinal lamina propria. Cross-species reactivity was preserved after elimination of somatic mutations and reversion to the germline configuration. In addition, unmutated Igs from a population of intestinal IgA-precursor B cells in PPs were found to be enriched for polyreactivity and cross-species reactivity relative to Igs produced by naïve B cells. This further suggested that subsets of circulating naïve B cells that are naturally poly- and cross-species-reactive are selected to become intestinal IgA precursors in PPs. Moreover, similar analyses performed with germ-free mice fed an antigen-free diet demonstrated that such selection occurs even in the absence of exogenous antigens. Added to the results of Chen et al. (2020) and Nowosad et al. (2020) regarding germ-free mice, these results further the notion that intrinsic processes may somehow select particular subsets of naïve B cells into PPs. In particular, Bunker et al. (2017) suggested the intriguing possibility that, in young mice, endogenous antigens may direct the selection of naïve B cells encoding Igs that can cross-react to a wide, but specific, subset of the GIT microbiota.

On the other hand, Kabbert et al. (2020) proposed a different mechanism for cross-species reactivity in the GIT microbiota, driven by affinity-matured SIgAs that have undergone somatic hypermutation. In their analyses, the binding capacity and spectrum of human adult SIgA was dependent on somatic mutations and did not correlate with polyreactivity. In this case, cross-species reactivity would likely be conferred by highly specific recognition of epitopes shared among different bacterial species. The authors suggested that the germline and affinity-matured SIgA responses could have a different weight at different life stages. In young individuals, antibodies have undergone fewer rounds of somatic mutation and are less affinity-matured (Lindner et al., 2015; Pabst and Slack, 2020). In this case, polyreactive, germline configuration antibodies could be the dominant mechanism of microbiota binding. With age, polyreactive antibodies could become replaced by affinity-matured antibodies that maintain cross-species reactivity through recognition of shared epitopes, in agreement with the fact that PC repertoires in the adult GIT are highly mutated (Barone et al., 2011; Benckert et al., 2011; Lindner et al., 2015).

### Possible Roles of SIgA-Microbiota Binding in the GIT

The development of experimental methods and animal models has demonstrated that SIgA pools are extremely heterogeneous, which, on top of the different mechanisms of SIgA binding, suggests a wide variety of potential biological functions for these antibodies. In the case of exogenous or opportunistic endogenous pathogens, that is, microbiota members that can overgrow and cause infections in immunocompromised or seriously ill individuals (Martin and Bachman, 2018), it is clear that SIgA coating contributes to their control by containing them spatially, interfering with their virulence mechanisms, minimizing their inflammatory potential, and/or facilitating their clearance from the GIT (Mantis et al., 2011). Pathogen clearance is enhanced by antibody-mediated agglutination, the clumping of cells in the presence of an antibody that binds multiple cells together. This clumping has been thought to occur upon bacterial collisions that allow cross-linking by the antibody. However, this classical agglutination model can only work when pathogen densities surpass 10^8^ bacteria per gram, which is not the case in typical infections in the gut lumen (Moor et al., 2017). Using a mouse model, Moor et al. (2017) showed that agglutination *in vivo* proceeds instead by a process that depends on bacterial growth rather than on cell collisions. In this enchained growth process, IgA-mediated cross-linking enchains daughter cells after division, preventing their separation and facilitating their clearance.

In the case of the non-pathogenic resident microbiota, the idea that the main function of SIgA-binding is promoting the elimination of microorganisms has been challenged (Fadlallah et al., 2018). Clearly, the fact that grams of SIgA bound to the microbiota are found in feces indicates that such interactions must have physiological relevance and could be required to maintain GIT homeostasis. Nevertheless, the functions of the various modes of SIgA-microbiota binding are currently debated and have not yet been clearly defined. Several not necessarily exclusive possibilities have been proposed. To some extent, SIgA serves the same function it displays in regards to exogenous pathogens, i.e., ensuring that microbiota members do not cross the epithelial barrier. Accordingly, bacteria of the Enterobacteriaceae family, which can often behave as opportunistic enteropathogens and are usually strongly bound by SIgA, do overgrow in the microbiota in cases of IgA deficiency (Fadlallah et al., 2018). But SIgA may also promote colonization by some bacterial species, although the extent to which this occurs and the mechanisms that may allow it are still unclear. Indeed, GIT microbiota alterations in people with IgA deficiency also include the lack of some species that are known to be normally coated by SIgA, and, overall, such species are more likely to be underrepresented than overrepresented in IgA deficiency. This is the case for common GIT Lachnospiraceae taxa such as *Coprococcus comes* and *Dorea* sp. (Moor et al., 2017; Fadlallah et al., 2018), contradicting the idea that SIgA binding always promotes bacterial clearance and demonstrating that it can rather have radically different effects depending on the bacterial species.

**Figure 1:**
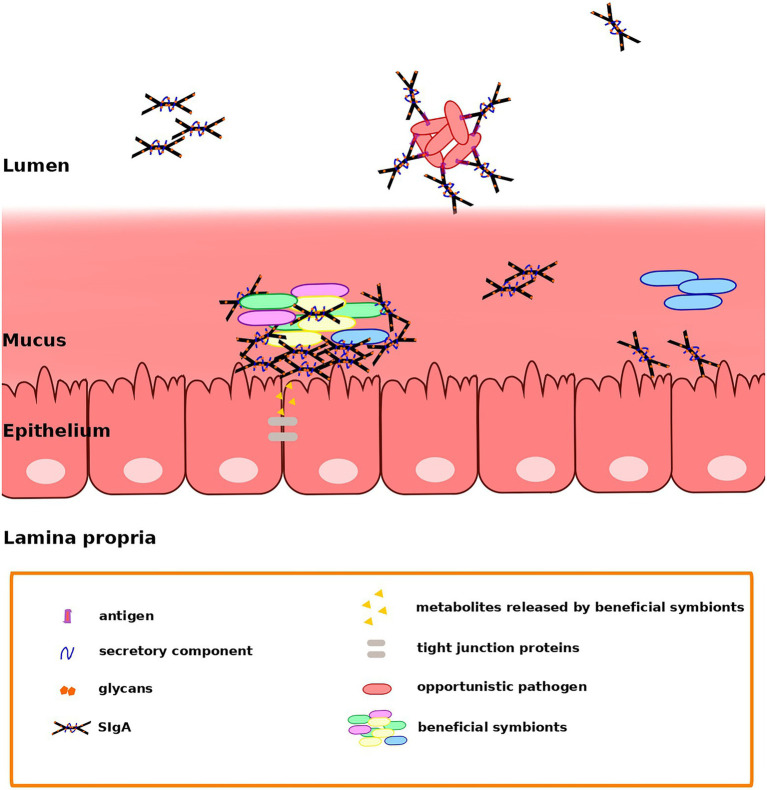
Secretory immunoglobulin A (SIgA) may favor the retention of beneficial members of the gut microbiota and the clearance of opportunistic pathogens through different modes of binding. Fab-dependent (canonical) binding may serve mainly to control microbiota members that may act as opportunistic pathogens, avoiding their penetration into the mucus. On the other hand, noncanonical binding may support the establishment of *Bacteroides* and Gram-positive symbionts in the mucus, maybe by contributing to the formation of a scaffold linking bacteria together and anchoring them to the underlying epithelium (Mathias and Corthésy, 2011). Nevertheless, direct interaction between epithelial cells and bacteria is prevented, avoiding an aggressive response by the immune system. Extracellular and intracellular receptors in epithelial cells recognize metabolites released by the gut microbiota, triggering the NFκB pathway and inducing an increased phosphorylation of tight junction proteins.

One possibility is that SIgA may enable colonization by promoting adhesion to the mucus layer that lines the GIT epithelium. This has been observed in the case of *Bacteroides fragilis*, in which complexes of multiple cells bound by SIgA have been detected in the mucus of the descending colon (Donaldson et al., 2018). *In vitro* results have demonstrated a better adhesion of SIgA-coated bacteria to monolayers of intestinal epithelial cells (IEC; Mathias et al., 2010). Further, it has also been proposed that SIgA may act as a scaffold to facilitate the formation of bacterial biofilms on the GIT mucosal surface although it remains controversial whether or not these biofilms exist. Electron microscopy does indicate the presence of “biofilm-like” structures on the GIT mucosa of rats, baboons, and humans (Palestrant et al., 2004). These biofilms may be supported by bacterially produced extracellular matrixes or by a mixture of host and microbial-derived components (Lee et al., 2011). In particular, *in vitro* models support the formation of bacterial biofilms containing both mucin (Macfarlane et al., 2005) and SIgA coating commensal bacteria (Bollinger et al., 2003, 2006; Orndorff et al., 2004). SIgA binding may contribute to biofilm formation through two non-mutually exclusive mechanisms: by forming a scaffold linking bacteria together or by anchoring the bacteria to the underlying epithelium (Mathias and Corthésy, 2011). Noncanonical binding of SIgA through Fab-independent complexes seems to be involved in the modulation of IEC responses and in the formation of the biofilms. Figure 1 illustrates potential modes of interaction among SIgA and beneficial symbionts or opportunistic pathogens of the gut microbiota, which may contribute to retention or exclusion, respectively.

In addition, noncanonical SIgA binding may contribute to promote colonization by certain microbiota species, not only by potentially facilitating retention, but also by providing nutrition. For instance, many *Bacteroides* strains are known to be able to metabolize mammalian N-glycans (Briliūtė et al., 2019) and could use the SIgA glycans involved in noncanonical binding as a carbon source. Indeed, it has been shown that the expression of polysaccharide utilization loci is induced in *B. thetaiotaomicron* upon noncanonical binding to SIgA through glycan interactions (Nakajima et al., 2018). However, Joglekar et al. (2019) have shown that IgA can also interfere with the utilization of polysaccharides. In a germ-free mouse model, they showed that colonization by *B. thetaiotaomicron* induced an IgA against proteins of the polysaccharide utilization locus that are necessary for utilization of fructan, an important dietary polysaccharide. Moreover, this resulted in downregulation of the expression of this locus. Therefore, IgA can also modulate bacterial growth in the GIT by regulating the utilization of important dietary microbiota-accessible carbohydrates (Joglekar et al., 2019).

### SIgA-Bacteria Interactions in the Context of Microbiota Development

The GIT microbiota develops in close interaction with immune and metabolic development, and with extensive variability across individuals depending on numerous factors, notably mode of birth and breast- or formula-feeding. Many works have explored microbial succession in the infant’s gut through metagenomic (Koenig et al., 2011; Vallès et al., 2014; Bäckhed et al., 2015) and metatranscriptomic sequencing (Gosalbes et al., 2019). These studies have shown that the GIT microbiota changes in composition and increases in species richness throughout the first year of life. After a first phase of microbiota adaptation to a milk-based diet, there is a marked change toward a more adult-like microbiota upon the introduction of solid foods and the cessation of breastfeeding (Vallès et al., 2014; Bäckhed et al., 2015; Fuertes et al., 2019). Whereas the early microbiota is enriched in genes facilitating lactate utilization, solid foods promote enrichment in genes coding for the utilization of a larger variety of carbohydrates, vitamin biosynthesis, and xenobiotic degradation. By 1 year of age, the microbiota of infants substantially resembles that of the adult, being dominated by the phyla Bacteroidetes and Firmicutes and by typically adult genera such as *Bacteroides*, *Faecalibacterium*, *Clostridium*, and *Ruminococcus*. Nevertheless, the microbiota keeps changing significantly during the first years of life (Yatsunenko et al., 2012) and differences from the adult pattern can still be detected even in adolescence (Hopkins et al., 2001; Agans et al., 2011).

Breast milk plays several complementary roles in shaping early gut microbiota and immune system development. These include prebiotic actions, by which nutritional components of human milk such as oligosaccharides (HMOs) select for the growth of specific types of bacteria, mainly *Bifidobacterium* (Coppa et al., 2004). Breast milk also transfers live bacteria to the infant (Martín et al., 2003; Cabrera-Rubio et al., 2012; Jost et al., 2013), as well as numerous factors that modulate and promote immune system development. These include IgAs, as well as antimicrobial compounds, immunoregulatory cytokines, and lymphocytes expressing gut-homing markers (Iyengar and Walker, 2012). The transfer of immunosuppressive and anti-inflammatory cytokines, such as IL-10 and TGF-β, as well as the promotion of IL-10 production in the infant, may facilitate the establishment of tolerance to the bacteria present in breast milk (Brandtzaeg, 2003; Dvorak, 2004). The microbiota promoted by breastfeeding also favors the development of the protective function of the gut mucosa immune system. In particular, the abundance of *B. infantis* during the first months of life has been shown to correlate with the levels of SIgA in the intestine (Sjögren et al., 2009).

Gut immunity undergoes substantial changes at weaning, as infants stop acquiring IgAs from their mothers and need to rely on IgAs of their own production (Gustafson et al., 2014). Planer et al. (2016) used IgA-seq profiles to study the development of gut mucosa IgA responses to members of the GIT microbiota in infants up to 2 years of age. They analyzed fecal samples collected in the first month of life and at 3-month intervals thereafter, and identified both IgA responses that were conserved across time as well as others that changed with age. Some taxa were consistently not targeted by IgA during the 2 years analyzed, including *Clostridium* species such as *C. clostridioforme* and *C. bolteae*. Others were consistently IgA-targeted only after the third month of life, including *C. nexile* and *B. bifidum*, while taxa such as *Ruminococcus torques* and *Akkermansia muciniphila* were targeted only from 6 to 24 months of age. The cohort was composed of twins, allowing assessment of the impact of family membership in this process: interestingly, the pattern of progression of IgA responses was highly distinctive for specific twin pairs during the first months of life and then generalized across all individuals in the second year. Mode of delivery and breastmilk or formula feeding also had an effect on the pattern of IgA targeting, notably with *E. coli* and *R. gnavus* being more and less targeted in breastfed infants, respectively. IgA-seq profiles also showed that the targeting of microbiota species by IgAs in 2-year-olds had already developed a pattern similar to that observed in their mothers (Planer et al., 2016).

**Figure 2:**
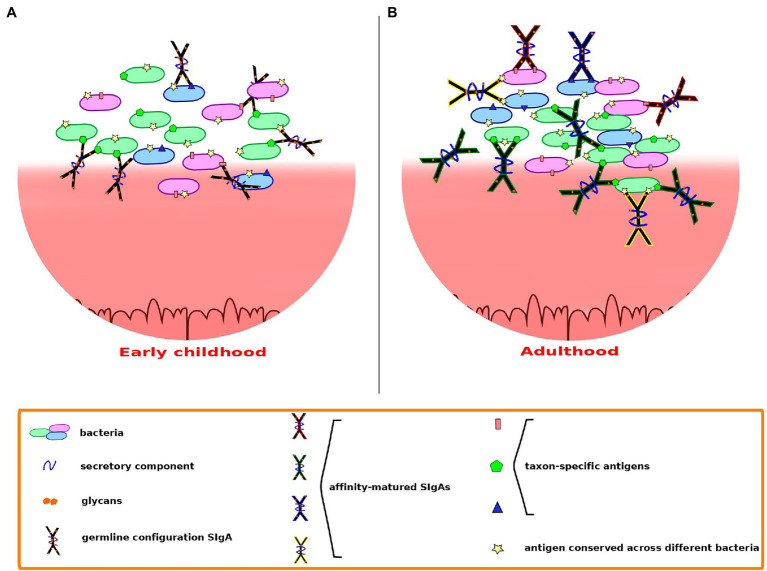
Secretory immunoglobulin A-microbiota interactions may differ depending on the host’s life stage. **(A)** In early childhood, possibly up to around 3 years of age, polyreactive SIgAs in germline configuration may dominate, cross-reacting through low to moderate affinity canonical binding. **(B)** In adults, somatic mutations undergone throughout life confer to SIgA the ability to recognize specific epitopes with high affinity, providing better recognition and containment of microbiota members that may act as opportunistic pathogens. Cross-species reactivity may continue through binding of conserved epitopes shared across species.

Beyond the specific patterns of bacterial targeting, the types of SIgA produced may also differ depending on the host’s life stage (Figure 2), as suggested by Pabst and Slack (2020) and Kabbert et al. (2020). As these authors point out, the generation of a wide collection of highly diversified and affinity-matured SIgAs likely requires a long period of time. Lindner et al. (2015) showed that, in both mice and humans, memory B cells established since early age are maintained and continuously diversified in subsequent years. This process is accompanied by dynamic changes in the pattern of somatic mutations, with their frequency increasing with age: in both mice and humans, the frequency distribution of IgA sequences shifted with age toward higher percentages of IgA sequences carrying high numbers of somatic mutations. The frequency of somatic mutations in 1–3-year-old children was similar to that observed in 12–23-week-old mice, and substantially lower than that of adolescents and adults. Further, in mice, B cell clones that persisted from 5 to 17 weeks of age were recovered and shown to carry increasing numbers of mutations. To explain this observation, Lindner et al. (2015) used photoconversion-based cell tracking to evidence that there are clones of gut PCs that derive from memory B cells. These cells can be recirculated to PPs and lymphoid follicles where they may undergo further hypermutation. This could result in an iterative process generating increased IgA specificity to the microbiota, in a manner akin to efficient vaccination responses, which are known to often require repeated entries of memory B cells into germinal centers (McHeyzer-Williams et al., 2015). Lindner et al. (2015) proposed that, in adults, adaptation of the IgA repertoire to the gut microbiota might thus derive mainly from the ongoing diversification of persistent B cell clones.

During the first years of life, the repertoire of IgA sequences present in the child is thus less diversified and affinity-matured than that of adults. Nevertheless, as the gut microbiota develops during this time to form and adult-like community, a large variety of incoming bacteria may reach and attempt to colonize the young child’s GIT, and the immune system needs to allow the establishment of beneficial bacteria while protecting the child from potential pathogens. The work of Bunker et al. (2017) indicates that most cross-reactive, low to moderate affinity IgAs produced by young mice preferentially target members of the Proteobacteria, which often are proinflammatory and can act as opportunistic pathogens. In contrast, these IgAs largely exclude taxa belonging to the Bacteroidetes and Firmicutes, to which many GIT symbiotic bacteria belong. Such bias suggests that coating by these early IgAs would serve to inhibit colonization rather than favor it. The low-affinity recognition of proteobacterial taxa in the young child’s GIT, possibly through the binding of their characteristic outer membrane molecules, such as LPS, may ensure a broadly directed containment of the potentially troublesome members of this phylum. Although high-affinity binding by T-cell-dependent, affinity-matured SIgAs seems to be required for the containment of *bona fide* Gram-negative enteric pathogens, low-affinity coating of proteobacterial taxa may still provide an advantage by decreasing their motility and their direct contact with the epithelial barrier or by interfering with their virulence mechanisms. Moreover, even the fast-breaking low-affinity bonds of T-independent SIgA coating may be sufficient to maintain enchained clumps of fast-growing, potentially pathogenic proteobacteria, facilitating their clearance (Hoces et al., 2020). The fact that many groups of jawed vertebrates do produce T-cell-independent, specialized mucosal antibodies suggests that low-affinity coating of GIT bacteria is evolutionarily advantageous (Mussmann et al., 1996; Zhang et al., 2010; Bunker et al., 2017), supporting the likely importance of this mechanism in young children before maturation of their adaptive immune capacities.

At the same time, the retention of beneficial symbionts that are crucial to sustain intestinal homeostasis in infants may be favored by noncanonical interactions with SIgA N-glycans. Beneficial Gram-positive species of the Firmicutes and Actinobacteria could be preferentially retained through binding of SIgA N-glycans to their peptidoglycan cell wall envelope, while prominent GIT Gram-negatives such as *Bacteroides* could be favored *via* utilization of the SIgA N-glycans as a source of carbon. The capacity of early SIgA pools to engage in a variety of asymmetrical interactions with different bacterial types could prime GIT microbiota assembly in the correct direction before T-cell dependent, affinity-matured responses become common in the gut lumen.

### SIgA-Microbiota Interactions in Disease

Investigations of potential alterations of IgA-bacterial binding have been a starting point to study different diseases, including Inflammatory Bowel Disease (IBD; Palm et al., 2014; Viladomiu et al., 2017; Kabbert et al., 2020; Rengarajan et al., 2020; Shapiro et al., 2021), postmenopausal breast cancer (Goedert et al., 2018), allergy (Dzidic et al., 2017), and pathogen infections (Džunková et al., 2016).

Palm et al. (2014) found that high coating by IgA marked disease-driving members of the intestinal microbiota in mice and humans. They demonstrated that fecal bacteria from IBD patients with high IgA coating transplanted into germ-free mice conferred dramatic susceptibility to colitis. In contrast, germ-free mice colonized with bacteria with low IgA coating showed minimal disease. However, in a recent work, Lima et al. (2022) were able to identify an IgA-coated *Odoribacter splanchnicus* strain whose transfer to ulcerative colitis patients through fecal microbiota transplantation (FMT) correlated with clinical improvement. The likely mechanism of action of this strain was investigated in mice with chemically induced colitis. In these, the *O. splanchnicus* strain was capable of inducing colonic Tregs and reducing weight loss and fecal lipocalin, a marker of ulcerative colitis (Stallhofer et al., 2015). The stimulation of IL-10 production by *O. splanchnicus* promoted Treg induction and limited the proliferation of Th17 cells, therefore reducing inflammation. Sort-chain fatty acids produced by *O. splanchnicus* also contributed to reduced weight loss and lipocalin levels through interactions with G protein-coupled receptors GPR43 and GPR109a, indicating that both immune and metabolic actions played a role in limiting colitis severity (Lima et al., 2022). On the other hand, Viladomiu et al. (2017) showed that Crohn’s disease patients that suffered from spondyloarthritis, a joint inflammatory disorder that commonly occurs as an extraintestinal manifestation in IBD, were enriched in IgA-coated *E. coli* that promoted Th17-dependent inflammation. In addition, Kabbert et al. (2020) showed that the IgAs of Crohn’s disease patients had a higher microbiota-binding capacity than those of healthy donors, binding a larger proportion of microbiota cells.

Džunková et al. (2016) observed in patients with *Clostridioides difficile* (formerly *Clostridium difficile*) infection (CDI) an increment of *C. difficile* clusters coated with IgA during the infection, while in the CDI-negative group *Fusobacterium* coating was predominant. Moreover, they detected that antibiotic treatment also influenced IgA responses to bacteria, increasing the coating of members of the order Lactobacillales, including *Enterococcus*. Nowadays, FMT is an effective treatment for CDI (Cammarota et al., 2015; Costello et al., 2015). Huus et al. (2021) observed in 48 recurrent *C. difficile* (rCDI) patients that IgA-bacterial interactions were modified compared with those in healthy persons, but FMT restored the interaction patterns of healthy donors. Before FMT, *E*. *coli* was the principal IgA-targeted taxon in rCDI, but after treatment *Ruminococcus* and *Dorea* were highly IgA-targeted as well.

In another study, Dzidic et al. (2017) showed that, when comparing children with allergic manifestations, principally asthma, vs. healthy children, the first group had a lower proportion of IgA bound to fecal bacteria at 12 months of age. Interestingly, this could not be associated with differences in IgA levels or bacterial load between the two groups. They also observed variability in the bacterial targets of early IgA responses and IgA recognition patterns between healthy children and children with allergic manifestations. At 12 months of age, *Escherichia/Shigella* species were predominantly IgA free in children with allergic manifestations, while *Lachnospiraceae* incertae sedis was mainly IgA bound. Goedert et al. (2018) suggested that the gut microbiota can be linked to increased breast cancer risk and indicated that breast cancer cases had significant oestrogen-independent associations with the IgA-coated and non-IgA-coated bacteria compared to controls. Women with breast cancer showed a reduced number of species in both IgA-positive and IgA-negative microbiota, with a more marked reduction in the IgA-coated fraction.

## Perspectives

The various lines of research explored in this review clearly indicate that the roles of SIgA in the GIT go beyond the exclusion of pathogens into a complex web of interactions with the resident microbiota. Future work will need to clarify how the various types of SIgA antibodies interact differentially with specific microbiota members, and how this variety of interactions results in homeostasis of the GIT ecosystem.

Several aspects of SIgA function revealed so far are particularly intriguing. Among these is the potential existence of intrinsic processes that select for particular subsets of naïve B cells into PPs, as suggested by the results in germ-free mice obtained by Bunker et al. (2017), Chen et al. (2020), and Nowosad et al. (2020). An interesting possibility is the proposal by Bunker et al. (2017) of an endogenous mechanism driving the selection of low-affinity IgAs that cross-react with a subset of the GIT microbiota enriched in potential opportunistic pathogens. Clearly, as discussed above, such capacity would be especially advantageous in young children whose T-cell-dependent responses are not yet matured. Therefore, natural selection could have driven the appearance of an innate mechanism that enables the systematic selection of early IgAs targeting common conserved extracellular elements present in microbiota members with high inflammatory and invasive potential. It will be crucial in upcoming years for experimental work to determine the extent to which this mechanism operates in humans at different life stages, as well as the basis for such innate IgA selection.

Furthermore, it will also be fundamental to continue investigating how SIgAs may favor the retention and/or growth of beneficial GIT symbionts. Current lines of evidence suggest that N-glycans and noncanonical binding are important players in this respect, but further work will need to clarify the mechanisms involved and their relevance *in vivo*. In particular, it will be important to understand the extent to which bacterial biofilms are present on the GIT mucosa and whether SIgA molecules play a role in facilitating their formation.

## Author Contributions

EDL and MPF conceived and wrote this review. All authors contributed to the article and approved the submitted version.

## Funding

MPF was supported by grant PID2019-105969GB-I00 (Ministry of Science and Innovation, Spain) during the preparation of this manuscript.

## Conflict of Interest

The authors declare that the research was conducted in the absence of any commercial or financial relationships that could be construed as a potential conflict of interest.

## Publisher’s Note

All claims expressed in this article are solely those of the authors and do not necessarily represent those of their affiliated organizations, or those of the publisher, the editors and the reviewers. Any product that may be evaluated in this article, or claim that may be made by its manufacturer, is not guaranteed or endorsed by the publisher.
