# Correction: Roles of secretory immunoglobulin A in host-microbiota interactions in the gut ecosystem

**DOI:** 10.3389/fmicb.2026.1868593

**Published:** 2026-05-20

**Authors:** E. Daniel León, M. Pilar Francino

**Affiliations:** 1 Department of Genomics and Health, Fundación Para el Fomento de la Investigación Sanitaria y Biomédica de la Comunitat Valenciana (FISABIO), Valencia, Spain; 2 CIBER en Epidemiología y Salud Pública, Madrid, Spain

**Keywords:** gut microbiota, microbiome, secretory IgA, immune system, cross-species reactivity

An incorrect **Funding** statement was provided. The correct funder is [MICIU/AEI /10.13039/501100011033]/the correct funding statement reads:

The author(s) declared that financial support was received for this work and/or its publication. MPF was supported by PID2019-105969GB-I00 funded by MICIU/AEI /10.13039/501100011033 during the preparation of this manuscript.

The original version of this article has been updated.

